# Micelle PCR reduces chimera formation in 16S rRNA profiling of complex microbial DNA mixtures

**DOI:** 10.1038/srep14181

**Published:** 2015-09-16

**Authors:** Stefan A. Boers, John P. Hays, Ruud Jansen

**Affiliations:** 1 Department of Medical Microbiology and Infectious Diseases, Erasmus University Medical Centre, Wytemaweg 80, 3015 CN, Rotterdam, The Netherlands; 2 Department of Molecular Biology, Regional Laboratory of Public Health Kennemerland, Boerhaavelaan 26, 2035 RC, Haarlem, The Netherlands

## Abstract

16S rRNA gene profiling has revolutionized the field of microbial ecology. Many researchers in various fields have embraced this technology to investigate bacterial compositions of samples derived from many different ecosystems. However, it is important to acknowledge the current limitations and drawbacks of 16S rRNA gene profiling. Although sample handling, DNA extraction methods and the choice of universal 16S rRNA gene PCR primers are well known factors that could seriously affect the final results of microbiota profiling studies, inevitable amplification artifacts, such as chimera formation and PCR competition, are seldom appreciated. Here we report on a novel micelle based amplification strategy, which overcomes these limitations via the clonal amplification of targeted DNA molecules. Our results show that micelle PCR drastically reduces chimera formation by a factor of 38 (1.5% vs. 56.9%) compared with traditional PCR, resulting in improved microbial diversity estimates. In addition, compartmentalization during micelle PCR prevents PCR competition due to unequal amplification rates of different 16S template molecules, generating robust and accurate 16S microbiota profiles required for comparative studies (e.g. longitudinal surveys).

Microbiota profiling methods are greatly enhancing our insights into the microbial diversity and taxonomy of many different types of environments and ecosystems, including the relationship between microbiota and host in health and disease^1^. The development of next-generation sequencing (NGS) technologies has highlighted the difficulties of assessing the microbiota using conventional culture methods, as PCR-based NGS of bacterial 16S rRNA genes yields a large diversity of 16S rRNA sequences that may be associated with a complex assortment of bacterial taxonomies – from phylum to genus level^2^. Although sequence-based approaches are incredibly powerful, it is important that scientists and bioinformaticians understand and acknowledge the current limitations and drawbacks of NGS technologies and appreciate that the choices made, from study design to DNA extraction and from DNA amplification to data analysis, can have serious impact on the microbiota profiles obtained^3^. For example, Kennedy *et al.* previously reported significant differences in DNA yield and bacterial DNA composition when comparing DNA extracted from the same fecal sample with different extraction kits^4^. In addition, the use of universal 16S rRNA gene PCR primers has led to inconsistencies in the literature regarding the abundance of the bacteria within similar ecosystems^5^. Essentially, the choice of the most optimal cell lysis procedures, and the most sensitive/specific universal 16S rRNA gene primer pair to be used, are greatly dependent on the sample type and target species to be investigated. Importantly however, even when using the correct choice of cell lysis procedure and 16S rRNA gene primer pair, amplification artifacts (chimeras) are inevitably generated during PCR amplifications due to the presence of multiple PCR targets in a single reaction chamber. Such chimeras are generated independent of the sample type used. Importantly, the formation of these chimeric sequences can lead to erroneous taxonomic identifications and overestimated microbiota richness^6^. Further, although chimeric sequences can be filtered out of NGS results using specialized software^7^,^8^, the generation of chimeric products can still seriously reduce the amount of useful information obtained in a single sequencing run^9^. Importantly, and this is seldom appreciated by users of NGS technologies, PCR is a competitive reaction meaning that the presence of multiple PCR targets in a single amplification reaction may lead to the preferential amplification of a particular subset of 16S rRNA gene copies^10^. The results could then be biased by factors related to the amplification efficiency of particular 16S rRNA amplicons rather than the relative abundance of 16S rRNA genes in the test sample. To overcome these sample-independent limitations, we developed and evaluated a micelle based amplification strategy targeting the 16S rRNA gene that greatly reduces chimera production during PCR amplification and prevents the formation of PCR competition products.

Micelle PCR (micPCR) is designed as a beadless emulsion PCR whereby a single molecule of template DNA is clonally amplified. Template DNA molecules are separated into a large number of physically distinct reaction compartments using water-in-oil emulsions. This compartmentalization per molecule reduces the probability of chimera formation and restrains PCR competition. For example, emulsion based amplification has been successfully applied for aptamer selection to reduce product-product and primer-product hybridizations^11^. Also, emulsion PCRs may be performed in BEAMing experiments, reliable and sensitive assays for the identification and quantification of variations in gene sequences and transcripts^12^. Finally, NGS platforms such as Ion Torrent (Life Technologies) and 454 (Roche) have adopted emulsion-based amplification strategies in their standard NGS workflows to clonally re-amplify DNA sequencing libraries, as their molecular detection methods are not sensitive enough for single molecule sequencing and to prevent mixed sequences.

## Results

**Figure 1:**
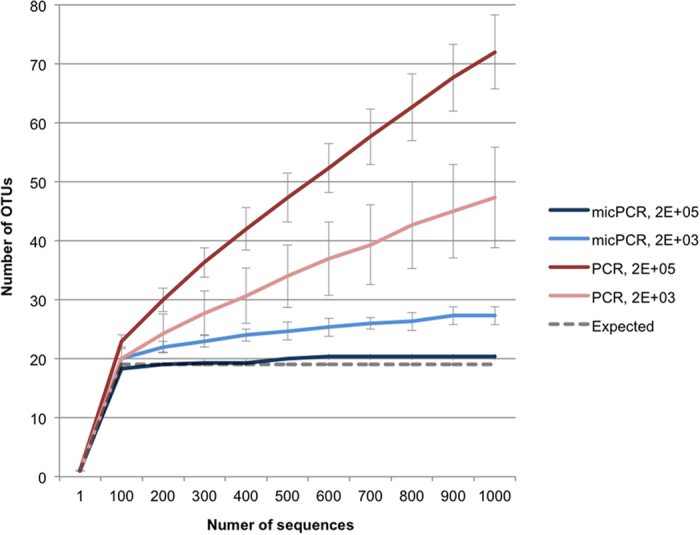
Comparison of rarefaction analyses between micPCR/NGS and traditional PCR/NGS using an equimolar, synthetic microbial community. The number of observed OTUs in the synthetic microbial community is shown as the function of the number of sequences obtained using micPCR/NGS reactions containing 2E + 05 (dark blue) and 2E + 03 (light blue) input molecules, and traditional PCR/NGS reactions containing 2E + 05 (dark red) and 2E + 03 input molecules (light red). Data points represent average values from triplicate experiments and error bars show standard deviations. Rarefaction curves were generated using mothur^19^ with an OTU defined at 97% similarity. Analysis was performed on a random 1,000-sequence subset from each sample. *Staphylococcus aureus* and *Staphylococcus epidermidis* present in the synthetic community could not be differentiated at a 97% similarity level, resulting in a maximum of 19 expected OTUs.

To evaluate the ability of micPCR to increase the accuracy of 16S rRNA sequencing, universal 357F and 926R primers were used to amplify the 16 S rRNA V3–V5 region from a synthetic microbial community containing equimolar 16S rRNA operon counts derived from 20 different bacterial species (HM-782D supplied by BEI Resources)^13^. The protocol utilized a two-step micPCR protocol, as well as a two-step traditional PCR protocol – used for comparative purposes – for NGS library preparation^14^. Importantly, the final number of amplification cycles of a two-step PCR protocol is higher compared to a one-step PCR protocol, resulting in an increased formation of chimeric sequences, making it suitable for evaluating the micPCR^15^. Results of triplicate experiments showed that micPCR/NGS generated only 1.5% (±1.2%) chimeric sequences in the synthetic community compared to 56.9% (±1.7%) chimeras using traditional PCR/NGS (Supplementary Table 1). For the micPCR/NGS, the rarefaction analysis rapidly reached horizontal equilibrium at the expected 20 operational taxonomic units (OTUs), indicating a highly reliable calculation of richness (Fig. 1). In contrast, the traditional PCR/NGS resulted in 72 OTUs in the synthetic community, with rarefaction analysis showing that the number of OTUs steadily increased as the number of sequence reads increased. It was found that the excess of OTUs consisted of chimeras of the sequences of the 20 species in the synthetic mix that had not been recognized as such by the Mothur software package (http://www.mothur.org/).

**Figure 2:**
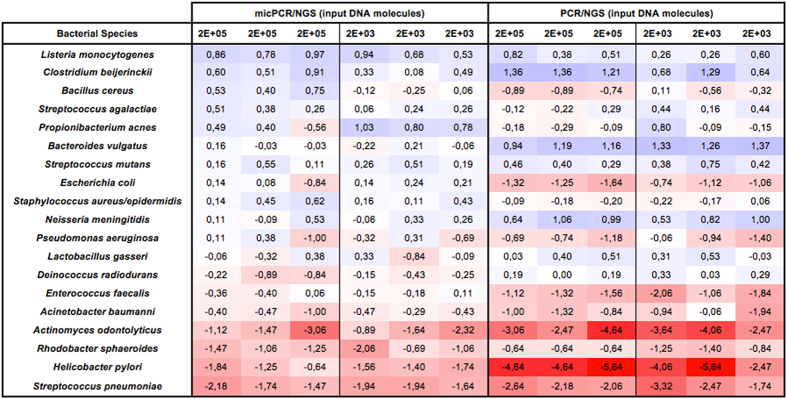
Quantitative accuracy of micPCR/NGS compared to traditional PCR/NGS from synthethic microbial community 16 S rRNA profiling. The observed species-level frequency data, corrected for the expected species-level frequency ratio for each of the synthetic community members, is shown as a heatmap using a binary logarithm scale. The expected frequency ratio is based on the reported equimolar 16 S rRNA operon counts derived from 20 bacterial species. Blue shades indicate an overestimation of species frequency and red colors an underestimation of species frequency. Data from triplicate experiments are presented individually.

Another important factor that influences NGS-related microbiota profiling is competition between different 16S rRNA molecules, resulting in unequal/preferential amplification rates for certain amplicon sequences. The result of competition can be an over- or underestimation of particular OTUs. For example, in our current experiments we utilized a synthetic community consisting of 20 bacterial species that are each present at an equimolar concentration of 5% of 16 S rRNA genes. MicPCR/NGS data showed an average 0.85-fold difference from the 5% OTU frequency expected in the synthetic community, with a maximum overestimation of 1.73-fold for *Listeria monocytogenes* and a maximum underestimation of 0.28-fold for *Streptococcus pneumoniae* (Fig. 2). In contrast, the OTU differences associated with PCR competition and traditional PCR/NGS were more extreme, yielding an average 0.65-fold difference in OTU frequency above the expected frequency, with an overestimated maximum of 2.31-fold for *Bacteriodes vulgatus* and an underestimated maximum of 0.04-fold for *Helicobacter pylori*. These findings are in agreement with the previously reported consistent overestimation of *Bacteriodes* spp. and underestimation of *Helicobacter* spp. in four different laboratories when investigating an identical synthetic community^13^.

In order to determine the usefulness of the micPCR/NGS protocol in determining the microbiota profiles of actual clinical and environmental samples, we evaluated the use of micPCR/NGS to determine the microbiota profiles for samples possessing a low diversity of bacteria (nose swabs), a medium bacterial diversity (feces), and samples containing a high diversity of bacteria (sludge). Results for three independent samples per sample type (nose, feces, sludge) revealed that chimeric sequences were reduced in all samples from an average of 38.0% (±15.7%) using traditional PCR/NGS to an average of 1.2% (±1.3%) using micPCR/NGS (Supplementary Tables 2–4). The reduction of chimera formation resulted in decreased richness values among all samples, particularly among the bacterially diverse feces and sludge specimens in which micPCR/NGS generated 212 (±30) OTUs less per 1,000 normalized sequences per sample than the traditional PCR/NGS protocol (Supplementary Figs. 1–3). In addition, differences were also observed in the quantitative OTU composition between individual clinical and environmental samples when comparing the micPCR/NGS results to the results obtained using traditional PCR/NGS. The maximum relative difference between identical OTUs within the same sample obtained by micPCR/NGS compared to traditional PCR/NGS was 17.0, 6.1, and 7.6% measured for the nose, feces, and sludge samples respectively (Supplementary Tables 5–7).

Finally, single molecule amplification using micPCR actually prevented the generation of chimeric products, due to the fact that we found an increase in chimeric sequences in the micPCR/NGS as the amount of template DNA molecules in PCR amplification reactions was increased (Supplementary Table 1). Importantly, the total template DNA molecules in a micelle PCR/NGS protocol should be kept below 10% of total micelle count to avoid any detectable chimera formation due to individual micelles hosting more than one template molecule. Therefore, the final numbers of target DNA molecules have to be carefully adjusted for each micPCR/NGS project to balance reaction yield and reaction specificity according to the experimental requirements.

## Discussion

In this report we show that the use of micelle PCR is particularly suitable for 16 S microbiota profiling experiments and strongly reduces the formation of the chimeric 16 S rRNA amplicons that are a major source of unidentifiable OTUs in microbiome studies. The authors developed and evaluated the use of a micelle based amplification strategy for 16S rRNA gene profiling of complex samples. Micelle or emulsion based amplification strategies have been successfully applied for a variety of DNA-targeted enzymatic reactions^11^,^17^. Most notably, Williams et published a protocol in 2006 describing the use of emulsion PCR to amplify complex gene libraries that reduce such amplification biases as chimeric sequences and competition between fragments of different lengths^17^. However, standardized commercial kits are now available to buy, which our micPCR protocol used, to offer a straightforward, easy and reproducible method to perform 16S rRNA micelle PCR.

Our results show that the use of micelle PCR/NGS greatly reduces chimera formation without the reliance on complex computational methods, resulting in improved microbial diversity estimates. An often-used approach to circumvent the overestimation of richness is to restrict analysis to OTUs that are found more than once, though the accompanying cost is a loss of sequencing sensitivity and accuracy due to the potential removal of singletons that are genuinely very low abundant representatives of their taxa within the total microbiome being profiled^16^. Further, it is true that the confidence of identifying: 1) truly low abundant OTUs and 2) singleton chimeric OTUs, increases as the number of sequence reads per sample is increased when using traditional PCR/NGS. This is because there is an increased chance of detecting multiple (>1) low abundant OTUs as the number of sequence reads increases. However, the researcher always has confidence that any singletons obtained using a micPCR/NGS protocol actually originate from low abundance bacterial species. This is because the number of chimeras formed using micPCR/NGS is very low and independent of the depth of sequencing.

The compartmentalization of template DNA molecules using micPCR/NGS prevents amplicon competition in PCR reactions, resulting in the generation of more accurate quantitative microbiota profiles. In addition to the standardized synthetic community experiments, different quantification values were also obtained from micPCR/NGS compared to traditional PCR/NGS performed on actual clinical and environmental samples. This results in different interpretations of sample composition and inter-sample variation. For example, micPCR/NGS showed a 3.3-fold reduction in *Staphylococcus* abundance among nose sample 1 compared to nose sample 3 (2.4% vs. 7.8%), whereas traditional PCR/NGS showed a 4.7-fold increase in *Staphylococcus* abundance among nose sample 1 compared to nose sample 3 (12.2% vs. 2.6%). Although the actual composition of these samples is unknown, the quantitative microbiota profiles obtained using micPCR/NGS likely represents a more accurate reflection of the true microbiota profiles as indicated previously using the synthetic community. Therefore, the use of micPCR/NGS will improve and help standardize microbiota profiling during comparative studies (e.g. longitudinal surveys). However, it should be noted that possible effects of sample handling, cell lysis and primer specificity on the final results of these microbiota profiles still exist. These factors should still be optimized for each type of test sample the researcher is investigating.

Taken together, our results show that micPCR/NGS increases the accuracy of 16S rRNA microbiota profiling when compared to traditional PCR/NGS, and its use should be recommended for future NGS projects due to the fact that chimera formation and PCR amplicon competition can potentially affect the accuracy of current microbiota profiling results.

## Methods

### Sample collection and DNA extraction

Genomic DNA from microbial mock community B (even, low concentration), v5.1 L, catalog no. HM-782D for 16 S rRNA microbiota profiling was obtained from BEI Resources, NIAID, NIH as part of the Human Microbiome Project and consists of genomic DNA from 20 bacterial strains with equimolar ribosomal RNA operon counts (100,000 copies per organism per μL). The microbial mock community contains species with different rRNA copy numbers in their genomes, ranging from two for *Helicobacter pylori* to 14 for *Clostridium beijerinckii*. Nose swabs and fecal samples were collected from healthy adult volunteers. DNA was extracted from both types of samples using the QIAsymphony instrument (Qiagen) according to the manufacturer’s instructions. DNA was extracted from three sludge samples from river bed, using the Powersoil DNA isolation kit (MO BIO Laboratories, Inc.). The total number of 16 S rRNA genes within each sample was quantified as described previously^18^. Prior to use as template for micelle and traditional PCR amplification the samples were normalized to 1E + 03 16 S rRNA genes/μL (nasal swabs) or 1E+05 16S rRNA genes/μL (feces and sludge samples).

### Micelle PCR amplification

The micPCR consisted of two PCR rounds of micPCR amplification. This was necessary, because micPCR only yields a limited number of amplicons per template molecule, which is a consequence of the limited reaction volume contained in a single micelle. We estimated that after a micPCR only 1E + 04 amplicon molecules were formed in a single micelle starting with a single genomic DNA fragment carrying a 16S rRNA gene copy. This low number of amplicon molecules is not sufficient for NGS of samples containing low amounts of bacterial DNA, such as nose swabs. However, using a second round of micPCR allowed us to increase the number of amplicon molecules for NGS, as well as allowing the addition of Molecular Identification sequences (MID) and Roche 454 specific A and B sequences. In the first step, micPCR was performed using modified 357F and 926R primers that amplified the V3–V5 regions of 16S rRNA genes and which incorporated universal sequence tails at their 5′ ends. In the second step, a micPCR was again used, but to amplify micPCR amplicons obtained from the first step micPCR. The second step micPCR utilized primers containing complementary sequences to the universal tails and included additional 454 sequencing-specific nucleotides, and specimen-specific MIDs. For both amplification steps, water-in-oil emulsions were prepared using the Micellula DNA Emulsion Kit (Roboklon). The oil phase comprised ~73% Emulsion component 1, ~7% Emulsion component 2, and 20% Emulsion component 3, which was mixed for 5 minutes in a cold room as described by the manufacturer. The aqueous phase was a PCR reaction mix comprising 0.01 mg/ml BSA, 2 μM of each primer, 200 μM dNTP mix, and 2.5 U Taq polymerase with 1× PCR Buffer B (EURx). Template DNA and water were added to give a final volume of 50 μL for each sample. Water-in-oil emulsions were prepared by adding 50 μL of pre-cooled PCR reaction mix to 300 μL of pre-cooled oil phase. The first round of micPCR was carried out using the following cycling conditions: 95 °C for 2 minutes followed by 25 cycles of PCR, with cycling conditions of 15 seconds at 95 °C, 30 seconds at 55 °C, and 60 seconds at 72 °C, and a final extension at 72 °C for 7 minutes. Emulsions were broken by the addition of 1 mL 2-butanol, and 400 μL of Orange-DX buffer (Roboklon) was added to the broken emulsion solution. This solution was centrifuged for phase separation. For the purification of DNA within the water phase, NucliSENS EasyMAG reagents (Biomérieux) were used according to the manufacturer’s instructions. To normalize DNA concentration and reduce the number of template molecules for the second round of amplification, the purified DNA was diluted 1E + 04 or 1E + 02-fold for high and low inputs, respectively, during the first micPCR. The second round of micPCR was performed under the following conditions: initial denaturation at 95 °C for 2 minutes followed by 25 cycles of PCR, with cycling conditions of 15 seconds at 95 °C, 30 seconds at 50 °C and 60 seconds at 72 °C. During the first 10 cycles of PCR, the annealing temperature was increased by 0.5 °C per cycle to an annealing temperature of 55 °C. The PCR was stopped after a final extension at 72 °C for 7 minutes. Again, emulsions were broken using 2-butanol, and DNA was purified using NucliSENS EasyMAG reagents (Biomérieux).

### Traditional PCR amplification

PCR reactions were performed in 10 μL volumes using the FastStart High Fidelity Reaction Kit (Roche) with the addition of 0.5 μM of each PCR primer. Resolight Dye (Roche) was added to measure DNA amplification in real-time using a LightCycler 480 instrument (Roche). The 16 S V3–V5 regions were amplified by PCR using modified 357F and 926R primers to allow for a two-step amplification strategy, using the following cycling conditions: initial denaturation at 95 °C for 2 minutes followed by 35 cycles of PCR, with cycling conditions of 30 seconds at 95 °C, 30 seconds at 55 °C, and 60 seconds at 72 °C. After PCR amplification, the amplicons were purified from unincorporated dNTPs, primers, primer dimers and salts using magnetic AMPure XP beads (Agencourt). The purified 16 S amplicons were re-amplified to incorporate 454 sequencing-specific nucleotides and specimen-specific MIDs. All PCR reactions were performed in 10 μL reaction volumes using the FastStart High Fidelity Reaction Kit with the addition of 0.5 μM of each PCR primer and the Resolight Dye. The PCR reactions were performed on a LightCycler 480 instrument, but under modified conditions: initial denaturation at 95 °C for 2 minutes followed by 35 cycles of PCR, with cycling conditions of 30 seconds at 95 °C, 30 seconds at 50 °C, and 60 seconds at 72 °C. During the first 10 cycles of PCR, the annealing temperature was increased by 0.5 °C per cycle to an annealing temperature of 55 °C. Bar-coded amplicons were mixed in equimolar concentrations and the complete pool was purified by gel extraction using the QIAquick Gel Extraction Kit (Qiagen), followed by a second purification with magnetic AMPure XP beads.

### Quantification of 16S molecules

In preparation for 454 sequencing (Roche), the concentration of purified amplicons obtained by micPCR and traditional PCR was measured using a 16 S quantitative PCR (qPCR). The qPCR reactions were performed in 10 μL reaction volumes using the LightCycler FastStart DNA Master SYBR Green I Kit (Roche) with the addition of 0.5 μM of amplification primer 357F and 926R without the universal tails. The PCR reactions were performed on a LightCycler 1.0 instrument (Roche), under the following conditions: initial denaturation at 95 °C for 10 minutes followed by 45 cycles of PCR, with cycling conditions of 1 second at 95 °C, 5 seconds at 55 °C, and 30 seconds at 72 °C. The concentration of purified amplicons obtained by micPCR and traditional PCR were normalized to 1E + 05 molecules/μL using a serial dilution of a standard solution containing 16S rRNA genes derived from a highly bacterial diverse sludge sample that was calibrated using the Quant-iT PicoGreen dsDNA Assay Kit (Life Technologies).

### Data Analysis

The composition of microbiota was determined by sequencing 16S rRNA genes using the 454 GS Junior Sequencer platform (Roche) according to the manufacturer’s instructions. NGS-data were automatically processed using the ‘Full Processing Amplicon’ pipeline available through the Run Wizard on the GS Junior Attendant PC (Roche). FASTA-formatted sequences were extracted from the .sff data file and processed using modules implemented in the Mothur v. 1.33.0 software platform^19^. Primer sequences were trimmed and sequences with length smaller than 400 were removed from the analysis. In addition, only the first 450 bases of each sequence were used for further analysis. In order to characterize the number of chimeric sequences more precisely, no additional quality filtering was applied. Unique sequences were aligned using the ‘align.seqs’ command and an adaptation of the Bacterial SILVA SEED database release 119 as a template (available at: http://www.mothur.org/wiki/ Silva_reference_alignment). Potentially chimeric sequences were detected and removed with the Uchime source code, using firstly the sequences as their own reference and sequentially the SILVA alignment version of the gold database (available at: http://www.mothur.org/wiki/ Silva_reference_alignment) as reference. The remaining aligned sequences were classified using a naïve Bayesian classifier with the SILVA SEED database release 119 and clustered into OTUs defined by 97% similarity. To reduce the effects of uneven sampling, all nose swab samples were rarefied to 500 sequences per sample and all other samples, including the synthetic community, feces, and sludge samples, were rarefied to 1000 sequences per sample. For all samples, rarefaction curves were plotted and the inverse Simpson’s diversity index and Good’s coverage were calculated. Finally, OTUs corresponding to the *Streptococcus* genus within the synthetic community were determined at species-level by checking the representative sequences against the reference sequences using Bionumerics version 5.10 (Applied Math).

## Additional Information

**How to cite this article**: Boers, S. A. *et al.* Micelle PCR reduces chimera formation in 16S rRNA profiling of complex microbial DNA mixtures. *Sci. Rep.* **5**, 14181; doi: 10.1038/srep14181 (2015).

## Supplementary Material

Supplementary Information
