# Supplementary material for: Micelle PCR reduces chimera formation in 16S rRNA profiling of complex microbial DNA mixtures
Source: Sci Rep. 2015 Sep 16;5:14181. doi: 10.1038/srep14181 (PMC4570986; doi:10.1038/srep14181)
Supplement: Supplementary Information [file srep14181-s1.doc]

**Micelle PCR reduces chimera formation in 16S rRNA profiling of complex microbial DNA mixtures**

Stefan A. Boers, John P. Hays, Ruud Jansen

**Supplementary Table 1. Sequence characteristics and diversity indices of a synthetic microbial community comparing the results of micPCR/NGS to traditional PCR/NGS.**

|  | **micPCR** | **micPCR** | **PCR** | **PCR** |
| --- | --- | --- | --- | --- |
| **Input molecules^a^** | 2E+05 | 2E+03 | 2E+05 | 2E+03 |
| **# sequences (range)^b^** | 3,161  (2,324 – 3,656) | 3,947  (1,310 – 7,185) | 3,718  (3,569 – 3,806) | 4,269  (1,692 – 5,607) |
| **% chimeras** | 1.5 (1.2) | 0.1 (0.2) | 56.9 (1.7) | 42.0 (5.5) |
| **# normalized sequences** | 1000 | 1000 | 1000 | 1000 |
| **# observed OTU’s** | 20 (1) | 27 (2) | 72 (6) | 47 (9) |
| **Inverse Simpson index** | 14.7 (1.2) | 16.0 (0.5) | 14.6 (0.4) | 14.1 (0.5) |
| **% Good’s Coverage** | 99.9 (0.0) | 99.4 (0.1) | 95.4 (0.4) | 97.5 (0.5) |

^a^ The number of allele copies of the 16S rRNA genes.

^b^ The number of sequences includes reads longer than 400 and shorter than 450 bases that were aligned using the SILVA alignment release 119 as reference.

The standard deviation is given in parentheses.

**Supplementary Table 2. Sequence characteristics and diversity indices of three nose swabs comparing the results of micPCR/NGS to traditional PCR/NGS.**

|  | **micPCR** | **PCR** | **micPCR** | **PCR** | **micPCR** | **PCR** |
| --- | --- | --- | --- | --- | --- | --- |
| **ID** | Nose 1 | Nose 1 | Nose 2 | Nose 2 | Nose 3 | Nose 3 |
| **Input molecules^a^** | 2E+03 | 2E+03 | 2E+03 | 2E+03 | 2E+03 | 2E+03 |
| **# sequences^b^** | 1,871 | 2,969 | 680 | 701 | 1,224 | 756 |
| **% chimeras** | 0.6 | 13.3 | 0.1 | 17.1 | 0.7 | 30.7 |
| **# normalized sequences** | 500 | 500 | 500 | 500 | 500 | 500 |
| **# observed OTU’s** | 19 | 21 | 13 | 13 | 22 | 31 |
| **Inverse Simpson index** | 1.8 | 2.7 | 1.6 | 1.6 | 2.5 | 2.0 |
| **% Good’s Coverage** | 98.8 | 98.8 | 98.8 | 98.4 | 98.2 | 96.4 |

^a^ The number of allele copies of the 16S rRNA genes.

^b^ The number of sequences includes reads longer than 400 and shorter than 450 bases that were aligned using the SILVA alignment release 119 as reference.

**Supplementary Table 3. Sequence characteristics and diversity indices of three feces samples comparing the results of micPCR/NGS to traditional PCR/NGS.**

|  | **micPCR** | **PCR** | **micPCR** | **PCR** | **micPCR** | **PCR** |
| --- | --- | --- | --- | --- | --- | --- |
| **ID** | Feces 1 | Feces 1 | Feces 2 | Feces 2 | Feces 3 | Feces 3 |
| **Input molecules^a^** | 2E+05 | 2E+05 | 2E+05 | 2E+05 | 2E+05 | 2E+05 |
| **# sequences^b^** | 2,532 | 3,325 | 2,052 | 4,605 | 1,390 | 4,435 |
| **% chimeras** | 0.6 | 43.8 | 4.5 | 58.5 | 1.7 | 57.0 |
| **# normalized sequences** | 1000 | 1000 | 1000 | 1000 | 1000 | 1000 |
| **# observed OTU’s** | 154 | 401 | 122 | 342 | 113 | 283 |
| **Inverse Simpson index** | 35.3 | 53.1 | 23.6 | 24.4 | 23.7 | 21.0 |
| **% Good’s Coverage** | 94.2 | 67.8 | 96.3 | 74.9 | 95.3 | 78.9 |

^a^ The number of allele copies of the 16S rRNA genes.

^b^ The number of sequences includes reads longer than 400 and shorter than 450 bases that were aligned using the SILVA alignment release 119 as reference.

**Supplementary Table 4. Sequence characteristics and diversity indices of three sludge samples comparing the results of micPCR/NGS to traditional PCR/NGS.**

|  | **micPCR** | **PCR** | **micPCR** | **PCR** | **micPCR** | **PCR** |
| --- | --- | --- | --- | --- | --- | --- |
| **ID** | Sludge 1 | Sludge 1 | Sludge 2 | Sludge 2 | Sludge 3 | Sludge 3 |
| **Input molecules^a^** | 2E+05 | 2E+05 | 2E+05 | 2E+05 | 2E+05 | 2E+05 |
| **# sequences^b^** | 2,399 | 3,470 | 1,734 | 2,985 | 2,284 | 2,676 |
| **% chimeras** | 0.4 | 46.2 | 0.9 | 39.0 | 1.2 | 36.8 |
| **# normalized sequences** | 1000 | 1000 | 1000 | 1000 | 1000 | 1000 |
| **# observed OTU’s** | 248 | 491 | 181 | 371 | 194 | 394 |
| **Inverse Simpson index** | 26.7 | 37.2 | 14.1 | 23.9 | 15.2 | 22.6 |
| **% Good’s Coverage** | 85.5 | 59.8 | 90.4 | 69.9 | 89.7 | 67.8 |

^a^ The number of allele copies of the 16S rRNA genes.

^b^ The number of sequences includes reads longer than 400 and shorter than 450 bases that were aligned using the SILVA alignment release 119 as reference.

**Supplementary Table 5. Relative OTU abundance within three nose swabs comparing the results of micPCR/NGS to traditional PCR/NGS.**

|  |  | **Nose 1** | | **Nose 2** | | **Nose 3** | |
| --- | --- | --- | --- | --- | --- | --- | --- |
| **OTU#** | **Taxon** | **micPCR** | **PCR** | **micPCR** | **PCR** | **micPCR** | **PCR** |
| 01 | *Corynebacterium* | 74.8% | 58.0% | 6.2% | 4.4% | 61.6% | 69.2% |
| 02 | *Peptoniphilus* | 5.8% | 4.0% | ND | ND | ND | ND |
| 03 | *Corynebacterium* | 4.0% | 1.8% | 10.2% | 10.8% | 7.4% | 8.8% |
| 04 | *Anaerococcus* | 3.2% | 11.4% | ND | ND | ND | ND |
| 05 | *Propionibacterium* | 2.6% | <1.0% | 3.8% | 3.4% | 7.2% | 5.8% |
| 06 | *Staphylococcus* | 2.4% | 12.2% | <1.0% | <1.0% | 7.8% | 2.6% |
| 07 | *Finegoldia* | 2.0% | 4.8% | ND | ND | ND | <1.0% |
| 08 | *Dolosigranulum* | 2.0% | 1.0% | <1.0% | ND | 5.8% | <1.0% |
| 09 | *Anaerococcus* | <1.0% | 2.4% | ND | ND | ND | ND |
| 10 | *Moraxella* | ND | ND | 77.0% | 79.0% | ND | ND |
| 11 | *Leuconostoc* | ND | ND | ND | ND | 4.0% | <1.0% |
| 12 | *Chloroplast* | ND | ND | ND | ND | 1,4% | 5.6% |
| 13 | *Lactococcus* | ND | ND | ND | ND | <1.0% | 1.0% |
| Others (<1%) | | 3.2% | 4.4% | 2.8% | 2.4% | 4.8% | 7.0% |

The lowest level of taxa classified with a Bayesian classifier using SILVA reference and taxonomic outlines (release 119) is presented. A cut-off of 1% abundance was used for visual differentiation between both techniques. ND = not detected.

**Supplementary Table 6. Relative OTU abundance within three feces samples comparing the results of micPCR/NGS to traditional PCR/NGS.**

|  |  | **Feces 1** | | **Feces 2** | | **Feces 3** | |
| --- | --- | --- | --- | --- | --- | --- | --- |
| **OTU#** | **Taxon** | **micPCR** | **PCR** | **micPCR** | **PCR** | **micPCR** | **PCR** |
| 1 | *Succiniclasticum* | 7.4% | 5.3% | ND | ND | ND | ND |
| 2 | *Anaerostipes* | 7.1% | 3.7% | ND | ND | ND | ND |
| 3 | *Ruminococcaceae* | 5.7% | 4.1% | <1.0% | <1.0% | 1.5% | <1.0% |
| 4 | *Faecalibacterium* | 5.6% | 7.5% | 14.7% | 18.2% | 9.3% | 11.3% |
| 5 | *Ruminococcaceae* | 4.7% | 3.5% | 1.4% | 1.8% | 5.2% | 4.4% |
| 6 | *Succinivibrio* | 4.4% | 2.0% | ND | ND | ND | ND |
| 7 | *Anaerotruncus* | 3.4% | 3.1% | ND | ND | <1.0% | ND |
| 8 | *RC9_gut_group* | 3.0% | 5.0% | ND | ND | ND | ND |
| 9 | *Clostridiales* | 2.7% | 1.8% | <1.0% | ND | ND | ND |
| 10 | *Ruminococcaceae* | 2.4% | 1.4% | <1.0% | <1.0% | 1.3% | 1.5% |
| 11 | *Ruminococcaceae* | 2.3% | 1.4% | ND | ND | ND | ND |
| 12 | *Lachnospiraceae* | 2.3% | 1.6% | ND | ND | ND | ND |
| 13 | *Ruminococcaceae* | 3.6% | 2.4% | ND | ND | 5.6% | 5.6% |
| 14 | *Bacteroides* | 1.8% | 2.0% | 2.7% | 2.0% | 10.8% | 14.9% |
| 15 | *Bacteroides* | 1.8% | 1.6% | 1.3% | <1.0% | 3.9% | 1.9% |
| 16 | *Blautia* | 1.4% | ND | 3.6% | 1.4% | <1.0% | <1.0% |
| 17 | *Ruminococcaceae* | 1.4% | <1.0% | ND | ND | ND | ND |
| 18 | *Bacteroides* | 1.3% | <1.0% | <1.0% | <1.0% | 3.4% | 2.0% |
| 19 | *Sutterella* | 1.2% | <1.0% | ND | ND | ND | ND |
| 20 | *Synergistes* | 2.2% | 1,0% | ND | ND | ND | ND |
| 21 | *Ruminococcaceae* | 1.1% | <1.0% | ND | ND | ND | ND |
| 22 | *Lachnospiraceae* | 1.1% | <1.0% | ND | ND | <1.0% | <1.0% |
| 23 | *Lachnospiraceae* | <1.0% | <1.0% | 11.6% | 5.5% | 2.3% | <1.0% |
| 24 | *Anaerostipes* | ND | ND | 3.1% | 1.2% | <1.0% | ND |
| 25 | *Ruminococcus* | <1.0% | <1.0% | 3.1% | 2.5% | 2.0% | <1.0% |
| 26 | *Sutterella* | ND | ND | 2.9% | 2.7% | 5.7% | 4.7% |
| 27 | *Bacteroidales* | ND | ND | 2.1% | 1.3% | ND | ND |
| 28 | *Coprococcus* | <1.0% | ND | 1.9% | <1.0% | ND | ND |
| 29 | *Barnesiella* | <1.0% | <1.0% | 1.9% | 1.5% | ND | ND |
| 30 | *Subdoligranulum* | <1.0% | <1.0% | 1.8% | 1.7% | 3.8% | 4.2% |
| 31 | *Lachnospiraceae* | <1.0% | ND | 1.8% | 1.2% | ND | ND |
| 32 | *Lachnospira* | ND | ND | 2.7% | <1.0% | ND | ND |
| 33 | *Blautia* | ND | ND | 1.6% | <1.0% | <1.0% | <1.0% |
| 34 | *Lachnospiraceae* | ND | ND | 1.6% | <1.0% | ND | ND |
| 35 | *Ruminococcus* | ND | ND | 1.5% | 1.8% | ND | ND |
| 36 | *Lachnospiraceae* | ND | <1.0% | 1.5% | <1.0% | <1.0% | ND |
| 37 | *Lachnospiraceae* | ND | ND | 1.4% | <1.0% | ND | ND |
| 38 | *Ruminococcaceae* | <1.0% | <1.0% | 1.4% | <1.0% | <1.0% | <1.0% |
| 39 | *Asteroleplasma* | ND | ND | 1.2% | 2.1% | ND | ND |
| 40 | *Lachnospiraceae* | ND | <1.0% | 1.1% | 1,0% | <1.0% | ND |
| 41 | *RF9* | ND | ND | 1,0% | <1.0% | ND | ND |
| 42 | *Bacteria* | ND | ND | <1.0% | 1.3% | ND | ND |
| 43 | *Parabacteroides* | <1.0% | <1.0% | <1.0% | <1.0% | 6.2% | 1,9% |
| 44 | *Bacteroides* | <1.0% | <1.0% | ND | ND | 3.2% | 2,2% |
| 45 | *Alistipes* | ND | ND | <1.0% | <1.0% | 5.2% | 4,7% |
| 46 | *Dialister* | ND | ND | ND | ND | 2.4% | 2,7% |
| 47 | *Ruminococcaceae* | <1.0% | <1.0% | <1.0% | <1.0% | 1.6% | <1.0% |
| 48 | *Acidaminococcus* | ND | ND | ND | ND | 1.5% | <1.0% |
| 49 | *Ruminococcaceae* | ND | ND | <1.0% | ND | 1.2% | <1.0% |
| 50 | *Subdoligranulum* | <1.0% | <1.0% | <1.0% | <1.0% | 1.1% | <1.0% |
| 51 | *Ruminococcaceae* | <1.0% | <1.0% | <1.0% | <1.0% | 1.1% | <1.0% |

The lowest level of taxa classified with a Bayesian classifier using SILVA reference and taxonomic outlines (release 119) is presented. A cut-off of 1% abundance was used for visual differentiation between both techniques. ND = not detected.

**Supplementary Table 7. Relative OTU abundance within three sludge samples comparing the results of micPCR/NGS to traditional PCR/NGS.**

|  |  | **Sludge 1** | | **Sludge 2** | | **Sludge 3** | |
| --- | --- | --- | --- | --- | --- | --- | --- |
| **OTU#** | **Taxon** | **micPCR** | **PCR** | **micPCR** | **PCR** | **micPCR** | **PCR** |
| 1 | *Zoogloea* | 15.1% | 14.1% | 22.6% | 15.9% | 18.6% | 11.0% |
| 2 | *Dechloromonas* | 7.0% | 3.8% | 2.1% | 1.3% | 1.9% | <1.0% |
| 3 | *Nitrospira* | 4.8% | 4.3% | 3.5% | 3.2% | 2.8% | 3.2% |
| 4 | *Comamonadaceae* | 3.8% | 3.4% | 2.5% | 1.7% | 12.4% | 8.0% |
| 5 | *Fusibacter* | 3.7% | 2.3% | 10.3% | 7.6% | 12.1% | 15.4% |
| 6 | *Comamonadaceae* | 2.5% | 1.6% | 1.1% | <1.0% | 1.4% | 1.5% |
| 7 | *Hydrogenophaga* | 2.4% | 1.1% | 5.0% | 6.3% | 2.1% | 2.9% |
| 8 | *Anaerolineaceae* | 2.2% | 2.3% | 1.4% | 2.0% | 1.4% | <1.0% |
| 9 | *Rhizobiales* | 2.2% | 2.2% | 1.8% | <1.0% | 1.5% | <1.0% |
| 10 | *Gammaproteobacteria* | 2.0% | <1.0% | <1.0% | 1.5% | <1.0% | <1.0% |
| 11 | *Blastocatella* | 2.0% | 1.9% | 2.0% | 3.1% | 2.2% | 1.7% |
| 12 | *Desulfuromonadales* | 2.0% | <1.0% | 1.7% | <1.0% | 3.2% | 4.2% |
| 13 | *Propionibacterium* | 1.6% | <1.0% | <1.0% | ND | <1.0% | ND |
| 14 | *Acidobacteria* | 1.3% | <1.0% | 1.2% | <1.0% | <1.0% | <1.0% |
| 15 | *Bacteria* | 1.2% | <1.0% | 1.1% | 1.3% | <1.0% | <1.0% |
| 16 | *Proteobacteria* | 1.1% | <1.0% | 1.3% | <1.0% | <1.0% | <1.0% |
| 17 | *Betaproteobacteria* | 1.0% | <1.0% | <1.0% | <1.0% | <1.0% | <1.0% |
| 18 | *Rhodocyclaceae* | 1.0% | <1.0% | 1.3% | ND | <1.0% | <1.0% |
| 19 | *Rhodobacteraceae* | <1.0% | <1.0% | 4.4% | 5.7% | <1.0% | <1.0% |
| 20 | *Corynebacterium* | ND | <1.0% | 1.7% | ND | <1.0% | ND |
| 21 | *Pseudomonas* | <1.0% | <1.0% | 1.4% | <1.0% | 2.7% | <1.0% |
| 22 | *Planctomycetes* | <1.0% | <1.0% | 1.0% | <1.0% | ND | ND |

The lowest level of taxa classified with a Bayesian classifier using SILVA reference and taxonomic outlines (release 119) is presented. A cut-off of 1% abundance was used for visual differentiation between both techniques. ND = not detected.

**
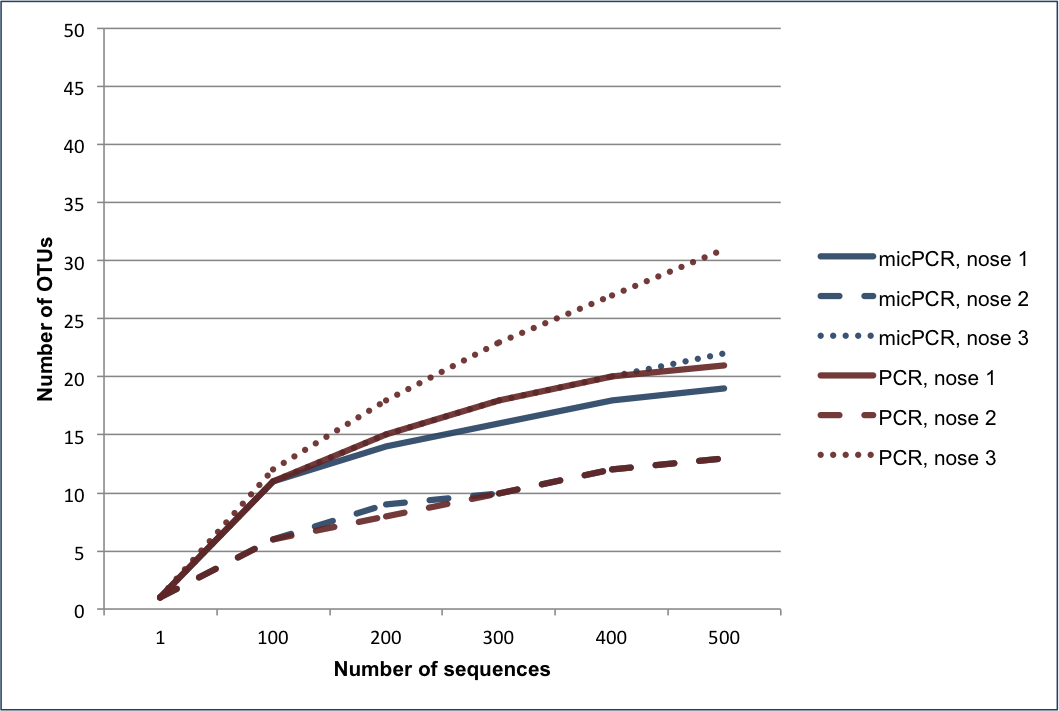
**

**Supplementary Figure 1.** Rarefaction analysis of three nose swabs comparing the results of micPCR/NGS to traditional PCR/NGS. The number of observed OTUs is shown as the function of the number of sequences, obtained using micelle PCR/NGS (blue) and traditional PCR/NGS (red). All rarefaction curves were generated using mothur, with an OTU defined at 97% similarity and analysis was performed on a random 1,000-sequence subset from each sample.

**
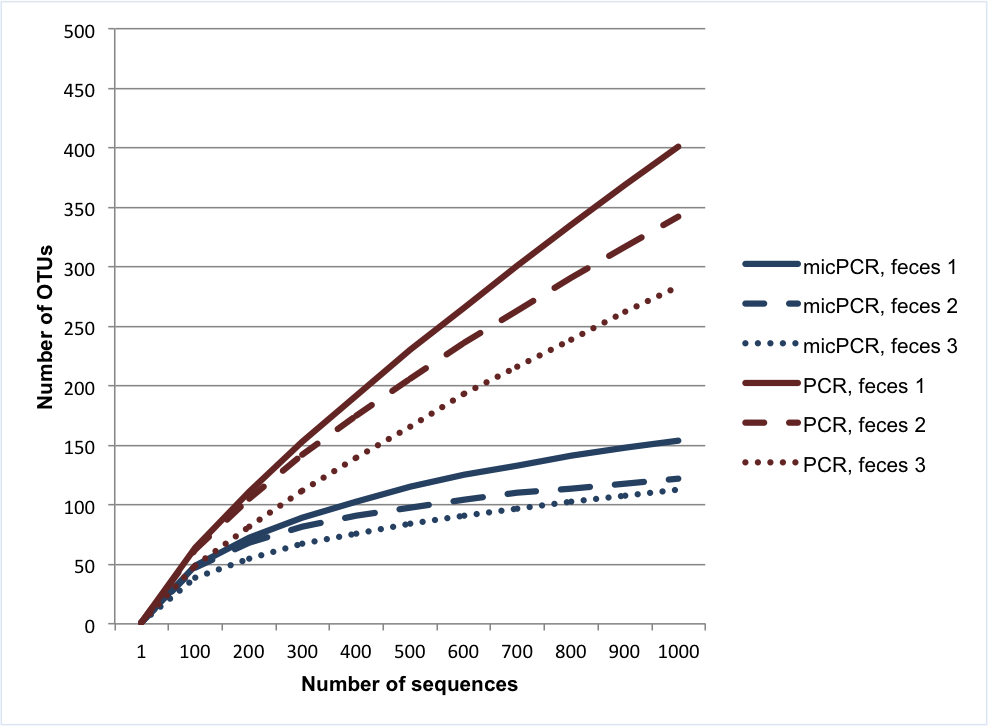
**

**Supplementary Figure 2.** Rarefaction analysis of three feces samples comparing the results of micPCR/NGS to traditional PCR/NGS. The number of observed OTUs is shown as the function of the number of sequences, obtained using micelle PCR/NGS (blue) and traditional PCR/NGS (red). All rarefaction curves were generated using mothur, with an OTU defined at 97% similarity and analysis was performed on a random 1,000-sequence subset from each sample.


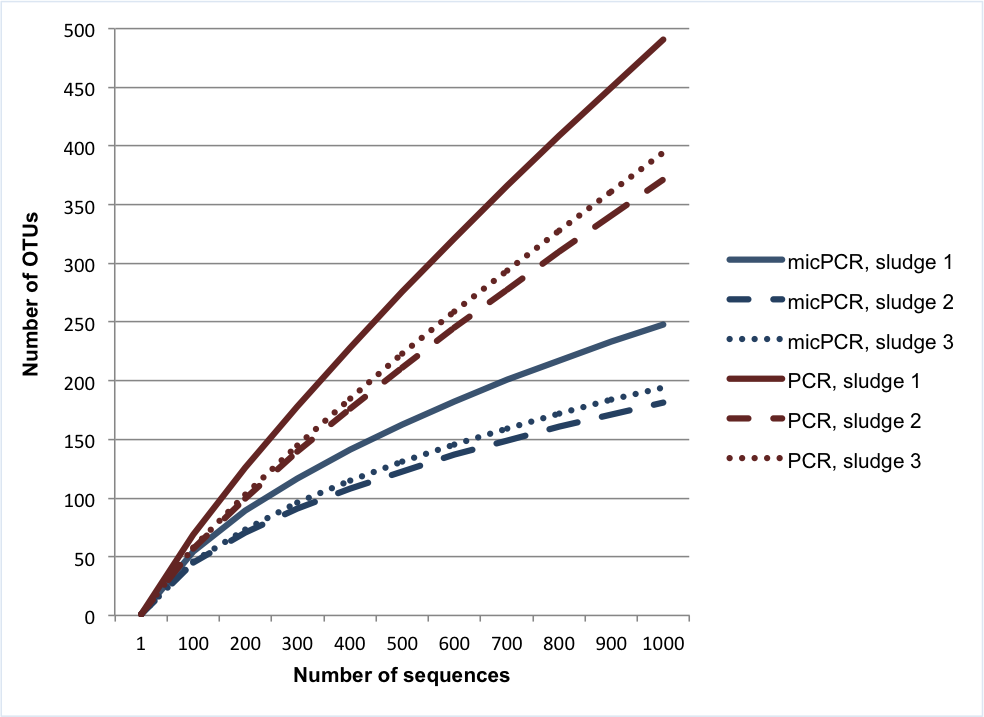


**Supplementary Figure 3.** Rarefaction analysis of three sludge samples comparing the results of micPCR/NGS to traditional PCR/NGS. The number of observed OTUs is shown as the function of the number of sequences, obtained using micelle PCR/NGS (blue) and traditional PCR/NGS (red). All rarefaction curves were generated using mothur, with an OTU defined at 97% similarity and analysis was performed on a random 1,000-sequence subset from each sample.
